# Development of a Low Mobility IEEE 802.15.4 Compliant VANET System for Urban Environments

**DOI:** 10.3390/s130607065

**Published:** 2013-05-29

**Authors:** Juan Antonio Nazabal, Francisco Falcone, Carlos Fernández-Valdivielso, Ignacio Raúl Matías

**Affiliations:** Electrical and Electronic Engineering Department, Public University of Navarre, Campus de Arrosadia 31006, Pamplona, Spain; E-Mails: francisco.falcone@unavarra.es (F.F.); carlos.fernandez@unavarra.es (C.F.-V.); natxo@unavarra.es (I.R.M.)

**Keywords:** Vehicular Ad-Hoc Networks (VANETs), IEEE 802.15.4, hybrid communication channel modeling

## Abstract

The use of Vehicular *Ad-Hoc* Networks (VANETs) is growing nowadays and it includes both roadside-to-vehicle communication (RVC) and inter-vehicle communication (IVC). The purpose of VANETs is to exchange useful information between vehicles and the roadside infrastructures for making an intelligent use of them. There are several possible applications for this technology like: emergency warning system for vehicles, cooperative adaptive cruise control or collision avoidance, among others. The objective of this work is to develop a VANET prototype system for urban environments using IEEE 802.15.4 compliant devices. Simulation-based values of the estimated signal strength and radio link quality values are obtained and compared with measurements in outdoor conditions to validate an implemented VANET system. The results confirm the possibility of implementing low cost vehicular communication networks operating at moderate vehicular speeds.

## 1. Introduction

**Figure 1.**
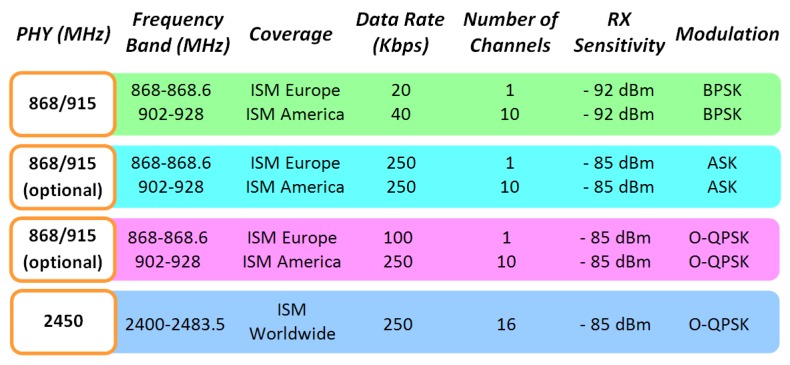
IEEE 802.15.4 characteristics.

The use of wireless communication systems is becoming widespread, with applications in virtually all domains, from domestic, industrial or security, to name but a few. One of the areas is which the possibility of seamless and ubiquitous operation is gaining interest is in vehicular communications, spanning from inter-vehicle, intra-vehicle and vehicle to infrastructure communications. In this sense, Vehicular *Ad-Hoc* Networks (VANETs) can provide useful functionality to enhance driver experience and warn from possible emergency situations present in highways. Nowadays the most popular VANET [1] implementations are based in the IEEE 802.11p [2] standard, specially designed for use in high mobility devices. If comparing with IEEE 801.11 standard [3], symbol time duration has been doubled for reducing the effect of Doppler spread and also guard time, for reducing Intersymbol Interference (ICI). This standard has two main drawbacks. The first of them is that it uses the licensed ITS band of 5.9 GHz (5.85–5.925 GHz), whereas the second one is the significant power consumption of this kind of devices. In this work, the idea is to use an IEEE 802.15.4 based system, a technology that has low power consumption, for implementing a simple VANET. As can be seen in Figure 1, the IEEE 802.15.4 standard [4,5] has different physical layers with different radio electric characteristics, depending on the specific application and location.

The physical layer to be employed in this work corresponds to the 2.4 GHz non-licensed ISM band. The maximum data rate is of 250 Kbps, considerably lower than those achieved by IEEE 802.11devices, but due the small amounts of information to be exchanged, in principle this is not a relevant issue. Furthermore, IEEE 802.15.4 devices are not designed for high mobility, with the main focus placed on their application for quasi-static wireless sensor networks in diverse application scenarios. Therefore, the goal is to determine if these devices are capable of operating in vehicular urban environments, with typical maximum speed limitations of 50 km/h. The use of such a type of system, in contrast with other systems such as mobile systems or 802.11 WLAN devices, can have potential benefits, based on their low power consumption, the high number of elements which can be allocated within the same network and the high reconfigurability that such systems exhibit.

The system developed in this work is a VANET that implements the RVC using XBee Pro IEEE 802.15.4 compliant communication modules produced by Digi. The transmission RF power level of these wireless communication devices can be adjusted to a maximum default value of 18 dBm which will be the value used in the simulation. In outdoor line-of-sight environments the typical range achieved is about 1,600 m and in indoor/urban locations, about 90 m, considering a receiver sensitivity of approximately −100 dBm. The modules are available with a variety of antennas, such as whip antennas (1.5 dBi), dipole antennas (2.1 dBi), low-profile chip antennas (−1.5 dBi) or a connector to which an external antenna can be connected. In this work, the antenna used was a chip antenna, that provides advantages in its form factor but however, it typically yields shorter range than the whip and dipole antenna options when transmitting outdoors. The proposed system potentially monitors the identification number of the vehicle as well as the values of the sensors placed on it. Doing this, the system knows the area in where the vehicle is, useful for electronic parking payments, electronic toll, road management control, to name but a few. Depending on the sensor placed on the vehicle, the system can also know the air pollution, noise pollution, exact GPS location, *etc*.

This work is structured as follows: Section 2 describes the simulation technique, based on a combined approach by means of simplified ray calculation coupled to time domain system level simulation; Section 3 describes measurement results for a VANET link established based on 802.15.4 standard. Finally, conclusions and future work lines will be presented.

## 2. Characterization of Vehicular Wireless Channel Properties

In order to gain insight into the performance of potential 802.15.4 devices in a VANET scenario, radiofrequency channel operation must be analyzed. Different approximations can be employed in order to analyze the characteristics of the wireless communication within a wireless vehicular scenario, from empirical-statistical methods to deterministic methods such as ray launching or full wave simulation techniques. These methods have been successfully applied to characterize complex scenarios [6,7], however require greater computational complexity to evaluate a large vehicular scenario. Therefore, in order to gain initial insight in the operation in a conventional environment while reducing computational complexity, an analytical approach of radiopropagation losses coupled with system level simulation techniques will be applied in this work. Power loss due to fading, as well as effects due to vehicular movement (*i.e.*, Doppler shift and Doppler spread) will be obtained in order to characterize the performance of such 802.15.4 based VANETs, as will now be described.

As previously stated, different aspects of radio electric propagation must be considered and simulated. The most important of them all is the Free-Space Path Loss (FSPL) [8]. When the radio electric signal propagates, its electromagnetic energy spreads, as an initial approximation, by the inverse square law, having lower energy densities for higher distances from the source. This effect is usually represented as a loss factor and calculated using Equation (1), which is only accurate in the far field [6]:

(1) $$\text{FSPL}(dB)=20\log_{10}(d)+20\log_{10}(f)-27.55$$

where ‘d’ is the distance in meters and ‘f’ is the frequency in MHz.

For antennas shorter than half of the wavelength of the radiation signal, the far field is the region for which the distance between antennas is much greater than twice the operating signal wavelength. Therefore, for an operating frequency of 2.4 GHz, we consider the far field region distances greater than 0.25 m. Another aspect to consider is the frequency shift due to Doppler effect, which consists of a relative shift of the frequency of operation due the relative speed between the emitter and the receiver, a situation that will occur when considering vehicular communication. The potential impact in communication system performance is degradation due to loss of synchronization in coherent detection receivers. The observed frequency ‘f’ is given by the following expression:

(2) $$f=\left(\frac{\nu \pm \nu_{r}}{\nu \pm \nu_{s}}\right)\;f_{0}$$

where ‘f_0_’ is the emitted frequency, ‘v’ is the wave propagation speed in the medium, ‘v_s_’ is the speed of the source or emitter in relation to the medium and ‘v_r_’ is the receiver speed in relation to the medium. In communication terms, it can be stated that the signal spectrum suffers a frequency shift. If the vehicle varies its velocity, the associated Doppler shift will vary as well, which may result in a random signal modulation. If the receiver is not moving and is the transmitter who moves in relation to it the Doppler shift associated is given by:

(3) $$f_{d}=\frac{\nu }{\lambda }\cos\theta$$

where ‘v’ is the transmitter speed, ‘λ’ is the signal wavelength and ‘θ’ is the angle that transmitter's movement direction forms in relation to the receiver. Therefore, by applying Equation (3), the maximum Doppler shift for a maximum vehicular speed of 60 km/h and a frequency of 2.4 GHz is approximately 133 Hz. If comparing this value with the 2 MHz bandwidth of an IEEE 802.15.4 channel, it can be supposed that the influence of the Doppler effect at this speed could be considered negligible. In multipath environments, when several reflected echoes of the original signal arrive at the receiver with approximately the same delay but different angle of arrivals or/and relative velocities, Doppler spread [1] appears. The received signal spectrum is a more noisy and spread version of the original signal, a combination of itself and the spectrums of the received echoes. Moreover, due to the fast variation rate of the wireless vehicular environment, the radio channel characteristics are not static and for quantifying this, Coherence Time term is defined. This term is the period of time that a channel may be approximated as time-invariant and it is inversely related to the Doppler Frequency calculated with Equation (2). For a working top speed of 60 km/h and a frequency of 2.4 GHz, the channel's Coherence Time is approximately of 75 ms. This Coherence Time is much longer than the time in which a typical IEEE 802.15.4 signal is transmitted, so it is reasonable to assume that the effect will be minimal.

**Figure 2.**
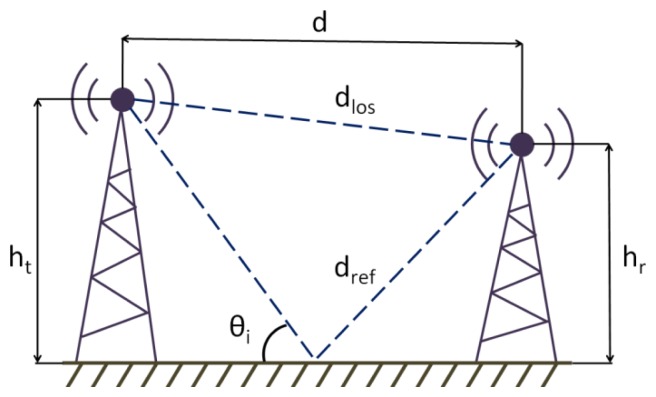
Two-Ray Ground Reflection model.

In order to simulate the vehicular wireless channel, we need to go beyond using a simple path loss estimation model like FSPL. More realistic treatment [9] of the path loss must consider that radio propagation will commonly suffer from, at least, one notable source of interference, as it is taken in consideration using the Two-Ray Ground Reflection model, illustrated in Figure 2.

The direct ray and the reflected one are received at the destination, mutually interfering due to the existence of destructive interference given by the relative phases of each vector component. The reflected ray has a phase difference with the direct one because the distance is greater, and it is also affected by the reflection coefficient due ground reflection [10–12]. This effect can be represented as a loss factor, given by the parameter L_tr_ and calculated using Equation (4), in which sub-variables for such equation are given in the expressions depicted in Equations (5)–(11):

(4) $$L_{\text{tr}}(dB)=20\log\;\left(4\pi \frac{d}{\lambda }{\left|1+\Gamma_{⊥}e^{i\varphi }\right|}^{-1}\right)$$

(5) $$\varphi =2\pi \frac{d_{\text{ref}}-d_{\text{los}}}{\lambda }$$

(6) $$\Gamma_{⊥}=\frac{\sin\theta_{i}-\sqrt{\in_{r}-\cos^{2}\theta_{i}}}{\sin\theta_{i}+\sqrt{\in_{r}-\cos^{2}\theta_{i}}}$$

(7) $$\in_{r}\;=\;\in_{r}-\;j60\sigma \lambda$$

(8) $$d_{\text{los}}=\sqrt{d^{2}+{\left(h_{t}-h_{r}\right)}^{2}}$$

(9) $$d_{\text{ref}}=\sqrt{d^{2}+{\left(h_{t}+h_{r}\right)}^{2}}$$

(10) $$\sin\theta_{i}=\frac{h_{t}+h_{r}}{d_{\text{ref}}}$$

(11) $$\cos\theta_{i}=\frac{d}{d_{\text{ref}}}$$

In the previous expressions, ε_r_ is the relative permittivity of the ground, σ the conductivity and *λ* the operating signal wavelength. If the transmitter is moving, because the direct ray and the reflected one reach the receiver with different angles of arrival, Doppler spread appears. A frequency shift due the Doppler spread must be added to the reflected ray, plus the attenuation and the phase shift due ground reflection.

**Figure 3.**
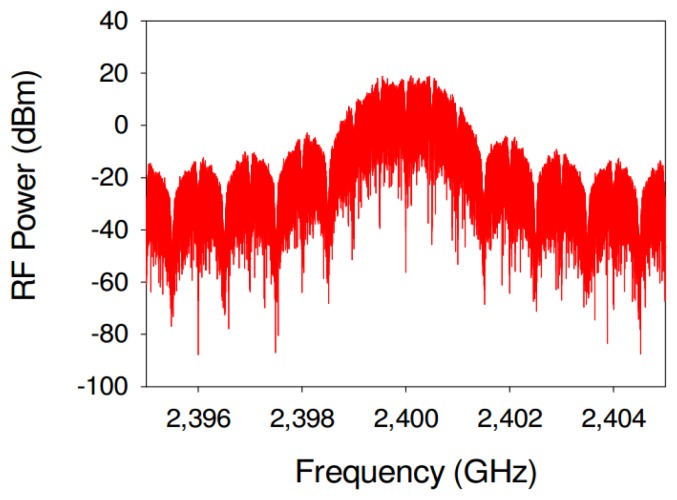
Simulated IEEE 802.15.4 spectrum, obtained with the aid of an in-house programmed module.

The material used in the simulation for the road surface is asphalt, and its electromagnetic parameters vary depending on the type and age of it. In this work, a relative permittivity of ε_r_ = 5, a conductivity of 0, a magnetic permeability of μ_r_ = 1 and IEEE 802.15.4 channel's central frequency of 2.4 GHz parameters have been used. The simulation has been developed using the Advanced Design System (ADS) [13] electronic design automation software for RF and microwave systems. This software has several different modules for simulating different RF technologies but not IEEE 802.15.4. Therefore, in order to use IEEE 802.15.4 in this work, specific modules have been developed in-house within our research group. The required custom Component Model has been implemented with the aid of the ADS Ptolemy Preprocessor Language in combination with standard C++ modules. Figure 3 shows a graphical representation of the simulated signal power spectrum. As can be seen, the shape of the spectrum consists on a main wider lobule and a periodic set of smaller lateral lobules, typical of ZigBee [14,15] signals and in general, physical layer implementations based on the IEEE 802.15.4 standard.

**Figure 4.**
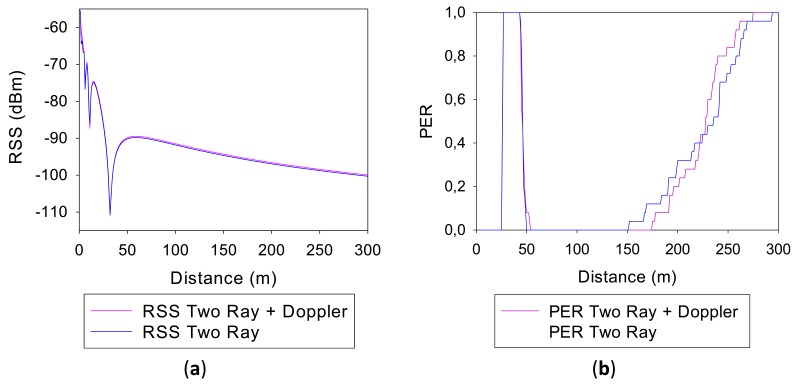
(a) RSS representation for antennas 1 m height; (b) PER representation for antennas 1 m height.

In order to determine the relevance that the height of the antennas has in the final results, two different simulations have been performed: one for a height of 1 meter and another one for a height of 2 m. Figure 4 shows respectively representations of the Received Signal Strength (RSS) and the Packet Error Rate (PER) values *versus* the distance from the source, both for antennas of 1 m height.

The values obtained using Two-Ray Ground Reflection models are shown in blue and the values obtained adding the Doppler Effect, in pink. It can be seen that for both simulation methods, the RSS representations are quite similar. There is a direct correlation between the received power levels and PER, where PER values increase as RSS values become lower, which is consistent with the limitations given by receiver sensitivity levels. Fading dips can be observed in specific distances, which are due to counter phase addition of direct as well as specular component in the two ray propagation model employed. It is worthwhile to note that these dips will be present in measurement results as well, but usually with lower rejection levels. This is given by the fact that in a realistic complex scenario the reflection coefficient will be lower than a perfectly reflecting surface, due to non ideal material properties as well as to surface roughness, which introduces diffuse scattering. Moreover, the real topology of the road is not completely flat, exhibiting a gradual change of height given by a small slope. This will modify the contact point of the reflected ray and therefore, the modulus and the phase of the observed field value in the receiver end.

**Figure 5.**
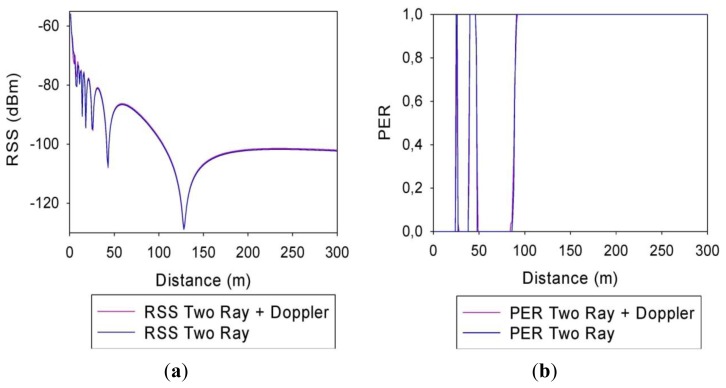
(**a**) RSS representation for antennas 2 m height; (**b**) PER representation for antennas 2 m height.

Figure 5 shows the same information that Figure 4 but for a simulation using antennas of 2 m height. It can be seen that, like in the other case and for both simulation methods, the RSS representations are almost equal. However, the selection of the antennas height is important in the final simulation results. This is due to the fact that the received levels will strongly depend on the impact point and angle of the incident and reflected rays, which will be given by the relative heights of the transmitter/receiver locations, consistent with the geometrical constraints and the vector nature of the propagated field components.

## 3. Implementation and Analysis of VANET Syste

**Figure 6.**
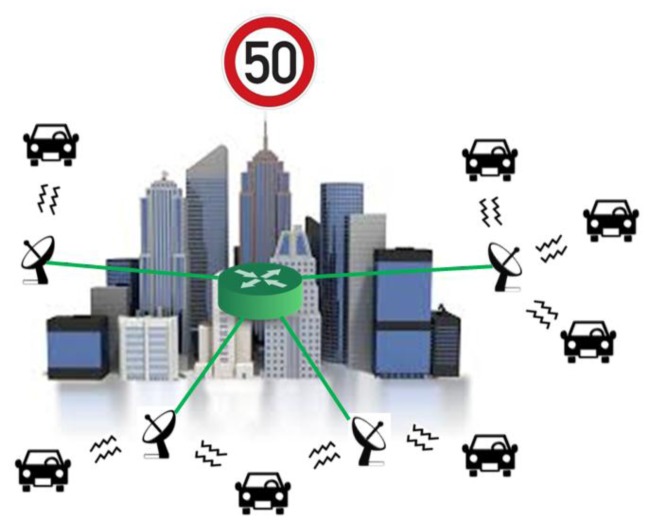
System topology of the proposed ZigBee network.

The VANET implemented in this work is a simple roadside-to-vehicle communication system with the idea of implementing a second inter-vehicle communication part in future developments. As it can be seen in Figure 6, the system consists in a series of static stations distributed along the zone to be monitored and that are interconnected between each other. Every static station sends a periodic broadcast signal asking for information to every vehicle that is within range and then waits for the response. When these stations obtain the vehicle data, they send this information to a central node for storing and subsequent processing. In this scenario, the main communication mechanism will be given by point to point radiolinks between the vehicles and the static stations.

**Figure 7.**
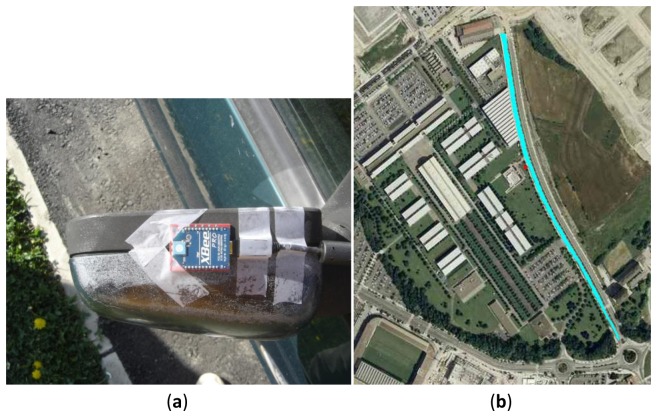
(**a**) Location of XBee Pro module at the rear view mirror position; (**b**) Image of the vehicle's trajectory in the measurement scenario.

In order to validate the simulation results obtained in the previous section, a series of field measurements have been performed on a VANET system that has been implemented for such a purpose. In the first place, a transmitting XBee Pro module has been placed the rear view mirror of a conventional sedan, as shown in Figure 7(a). On the other hand, a receiving XBee Pro module has been placed at the point represented with a small red dot in Figure 7(b). This figure shows an image of the measurement environment and the road that the vehicle will follow is depicted by a blue line, going from north to south direction. The heights of both antennas are similar and approximately of 1 m each. The transmitting XBee Pro module sends packets at maximum speed, each one of them with a unique identification number, which increases with every packet sent. When a packet arrives at the receiving XBee Pro module, its arrival time, identification number and RSS values are stored in a text file. This measurement setup is employed to characterize a point to point radiolink, which can be generalized to the case of multiple vehicles by considering adequate frequency channel allocation. In such case, which is necessary to guarantee adequate co-channel interference levels, multiple vehicle scenarios can be considered by aggregation of the individual radiolinks which are characterized. Additional interference levels can be considered by considering new power density levels, which can be obtained as equivalent Additive White Gaussian Noise sources included at receiver level.

**Figure 8.**
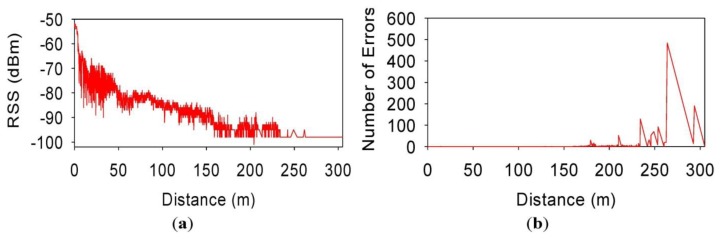
(**a**) Measured packet RSS values for 30 km/h speed; (**b**) Measured number of error packets for 30 km/h speed.

Figure 8 shows respectively representations of the Received Signal Strength (RSS) and the number of error packets *vs.* the distance from the source, for a speed of 30 km/h. If comparing RSS measured values with the simulated ones, it can be seen that the values are quite similar for virtually all distances. Comparison of simulated and measured results of Packet Error Rate values show good agreement, as can be seen from the previous figures. It can be seen at a distance of approximately 150 m from the transmitter, PER values begin to increase, as well the number of individual erroneous packets. It is worthwhile to note that multipath propagation effects and possible interferences with nearby wireless devices working in the same frequency band have been not taken in consideration in the simulation. That is the reason why field measurements are not exactly identical to the simulated ones, but they are close enough.

**Figure 9.**
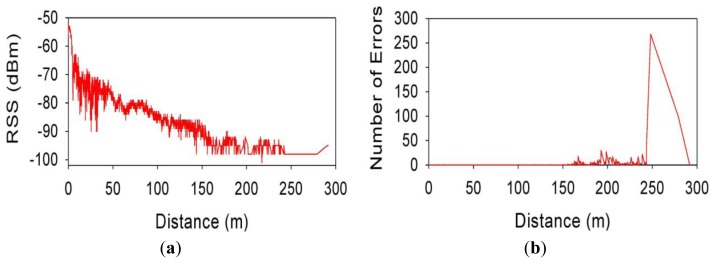
(**a**) Measured packet RSS values for 60 km/h speed; (**b**) Measured number of error packets for 60 km/h speed.

Figure 9 shows the same information that Figure 8 but for a vehicle speed of 60 km/h. It can be seen that these results are quite similar; indicating that for distances from the transmitter between 150 and 200–250 m, IEEE 802.15.4 can work with good radio link quality, for speeds of 30 km/h and 60 km/h.

**Figure 10.**
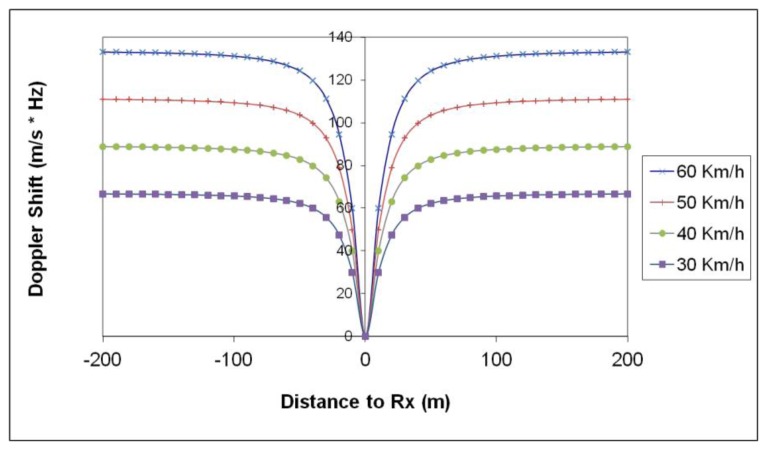
Doppler Shift as function of static TX and moving RX distance.

The proposed scenario describes a medium density urban environment, which is fairly constant in terms of slow fading characteristics, given by the topology and morphology and statistically described by the standard deviation of the propagation loss model employed. In order to gain more insight in the impact of low speed mobility, Doppler shift as well as speed variation in data transmission performance have been analyzed. Figure 10 depicts the simulation results obtained for the proposed scenario of the observed Doppler shift as a function of static transmitter and moving receiver distance. As the speed of the vehicle increases, the Doppler shift rises, within the order of 100 Hz, a value which can degrade operation as a consequence of loss of synchronism.

**Figure 11.**
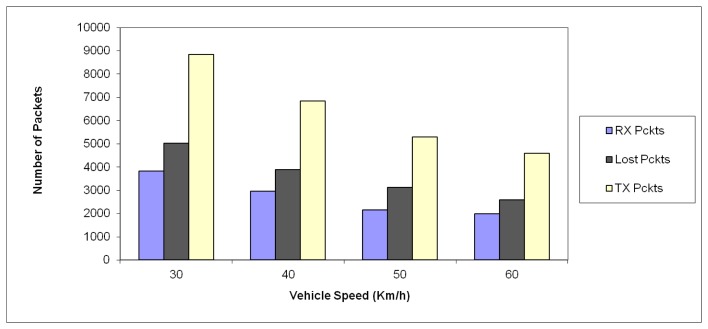
Variation in System Performance as a function of vehicle speed.

In order to account for the impact of change in vehicle speed, several measurement trials have been performed, with speeds from 30 km/h up to 60 km/h, as depicted in Figure 11. As it can be seen, as the speed is increased, the number of transmitted packets decreases, due to the fact that the observation time of the received decreases. The packet error ratio in all cases is similar, in the order of 40.7% to 43.3%. This result is determined by the sensitivity level of the wireless sensors.

Another step in the analysis of the performance of the proposed VANET system is to analyze its implementation at a system level, which will be described in this section. A receiver XBee Pro module is placed in a fixed static base station and a transmitting XBee Pro module is placed at every vehicle attached to the system. At a given time interval, the base station send periodically an information request packet to every vehicle that is in range. Each vehicle responds with a data packet that contains its identification number and the data registered by all available sensors.

**Figure 12.**
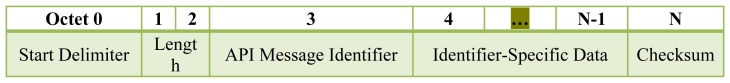
API Frame Structure.

The XBee Pro [16] modules are programmed and controlled using local and/or over-the-air Attention (AT) commands, usually employed in order to control modem operation. The modules can operate in Transparent Mode, in which they act as a serial line replacement or in Application Programming Interface (API) mode. The API operation option facilitates many operations, as RSS and payload information from received data packets. When in API mode, all data entering and leaving the module is contained in frames that define operations or events within the module. The structure of this kind of frames is shown in Figure 12.

**Figure 13.**
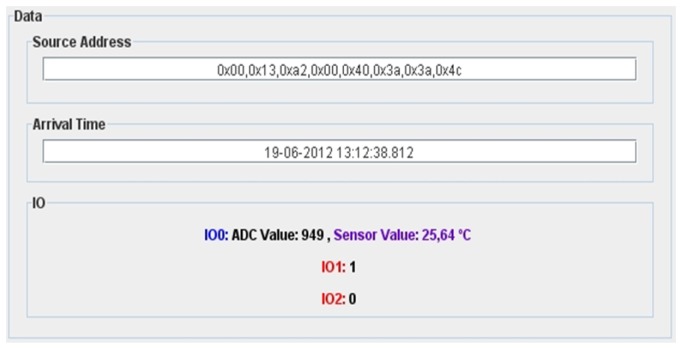
Screenshot of main control application GUI.

Every XBee Pro module has a unique read-only IEEE 64-bit address that unequivocally identifies it. It has also nine I/O pins that can be configured as digital output, digital input or analog input, the last one with an analog-to-digital (ADC) conversion of 10 bits. When a data packet is receiver, the information it contains is extracted and represented in the Graphical User Interface (GUI) on the main control application (see Figure 13). In the “Source Address” field, the vehicle identification number is shown as a hexadecimal array representing the eight bytes of the vehicle IEEE 64-bit address and in the “Arrival Time” field, the time and the date when the data packet was received is shown. Finally, in the I/O panel, a dynamic representation of all sensor data values contained in the data packets is shown. Analog data sensors are painted in blue and digital ones, in red. The analog data is represented as the ADC conversion level (between 0 and 1,023) and also the final measured value, depending on the sensor type. The received information is also stored in a MySQL database table. Every new entry has five different parameters:

- *idnodo*: This parameter consists of a eight byte size integer that stores the IEEE 64-bit address from where the packet has arrived.
- *timestamp*: This parameter consists in a eight byte size integer that stores the packets arrival time, represented by the number of milliseconds elapsed between the arrival time and the first of January of 1970, 00:00:00.000 GMT (Gregorian).
- *numsensores*: This parameter consists of a one byte size integer that stores the number of different sensors that are present in the data packet.
- *idsensores*: This parameter consists of a dynamic binary string that stores the type of the different sensors that are present in the data packet. The final value is the concatenation of every sensor type number represented as a one character size hexadecimal string.
- *valorsensores*: This parameter consists of a dynamic binary string that stores the type of different sensors that are present in the data packet. The final value is the concatenation of every sensor data value represented as an eight character size hexadecimal string.

**Figure 14.**
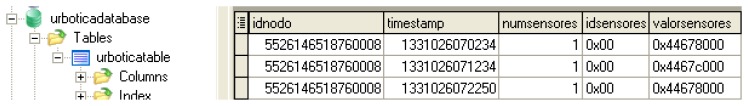
Screenshot from stored data.

For clarification, Figure 14 shows a screenshot taken with a MySQL client of the stored data of three received data packets. As can be appreciated, all of the packets have been received from the same vehicle that has one sensor only, and its value has changed between arrivals.

## 4. Conclusions

In this work, a VANET system implemented with the aid of 802.15.4 transceivers has been analyzed, simulated and measured. Simulation results have been obtained by coupling simplified analytical radiopropagation model fitted with a two ray multipath estimation with a system level 802.15.4 module implemented in-house. A vehicular scenario, consisting of a static transceiver and a transceiver mounted on a moving vehicle on a conventional urban road has been employed, with the aid of a VANET system implemented on the basis of XBee 802.15.4 modules. The results reveal that using IEEE 802.15.4 is suitable for moving vehicles when the maximum speed is in order of 60 km/h and with an operating range, depending on the antennas used and the receiver sensitivity values, of about 250 m. It can be also seen that the radio link characteristics does not significantly vary between speeds of 30 and 60 km/h.

A potential limitation in the use of IEEE 802.15.4 devices is that both transmitter and receiver must work in the same channel. This could be a problem if there are too many vehicles working at the same channel because they would interfere with each other and cause congestion. A possible solution for this issue could consists of installing at the base station one receiver module for every different channel and assigning vehicles channels randomly, for maximizing the use of the 2.4 GHz bands spectrum. Moreover, complex scenarios could be analyzed with the aid of deterministic radio channel methods, by increasing computational complexity. The use of 802.15.4 based VANET can aid in the implementation of low cost and low energy consumption systems for diverse uses in a vehicular scenario, such as traffic information, emergency situations or marketing purposes, among others.

## References

[1] Hartenstein H., Laberteaux K.P. (2010). VANET Vehicular Applications and Inter-Networking Technologies.

[2] IEEE Standard for Information Technology—Telecommunications and Information Exchange between Systems—Local and Metropolitan Area Networks Specific Requirements; Part 11: Wireless LAN Medium Access Control (MAC) and Physical Layer (PHY) Specifications, Amendment 6: Wireless Access in Vehicular Environments.

[3] IEEE Standard for Information Technology—Telecommunications and Information Exchange between Systems—Local and Metropolitan Area Networks Specific Requirements; Part 11: Wireless LAN Medium Access Control (MAC) and Physical Layer (PHY) Specifications.

[4] IEEE Standard for Information Technology—Telecommunications and Information Exchange between Systems—Local and Metropolitan Area Networks Specific Requirements; Part 15.4: Wireless Medium Access Control (MAC) and Physical Layer (PHY) Specifications for Low-Rate Wireless Personal Area Networks (WPANs).

[5] Yang X., Pan Y. (2009). Emerging Wireless LANs, Wireless PANs, and Wirelessmans: IEEE 802.11, IEEE 802.15, IEEE802.16 Wireless Standard Family.

[6] Azpilicueta L., Falcone F., Astráin J.J., Villadangos J., García Z.I.J., Landaluce H., Angulo I., Perallos A. (2012). Measurement and modeling of a UHF-RFID system in a metallic closed vehicle. Microwave Opt. Technol. Lett..

[7] Nazábal J.A., Iturri L.P., Azpilicueta L., Falcone F., Fernández-Valdivielso C. (2012). Performance analysis of IEEE 802.15.4 compliant wireless devices for heterogeneous indoor home automation environments. Int. J. Antennas Propagat..

[8] Freeman R. (2007). Radio System Design for Telecommunication.

[9] Giordano E., Frank R., Ghosh A., Pau G.G.M. Two Ray or not Two Ray This is the Price to Pay.

[10] Sommer C., Dressler F. Using the Right Two-Ray Model? A Measurement-Based Evaluation of PHY Models in VANETs.

[11] Rappaport T.S. (2009). Wireless Communications: Principles and Practice.

[12] Xia H., Bertoni H. (1993). Radio propagation characteristics for line-of-sight microcellular and personal communications. IEEE Trans. Antennas Propagat..

[13] Advanced Design System (ADS). www.home.agilent.com/en/pc-1297113/advanced-design-system-ads.

[14] Farahani S. (2008). Zigbee Wireless Networks and Transceivers.

[15] Dargie W., Poellabauer C. (2010). Fundamental of Wireless Sensor Networks: Theory and Practice.

[16] Product Manual v1.xEx—802.15.4 Protocol, Digi International, 2013. http://www.digi.com/pdf/ds_xbeemultipointmodules.pdf.

